# Identifying students of concern. Primary school teachers’ reflections on assessing students’ general school functioning based on the concept of typicality

**DOI:** 10.3389/fpsyg.2025.1601780

**Published:** 2025-08-04

**Authors:** Stine Ekornes, Solveig Holen, Jo Magne Ingul, Simon-Peter Neumer, Frode Adolfsen, Elisabeth Valmyr Bania

**Affiliations:** ^1^Regional Centre for Child and Youth Mental Health and Child Welfare, Norwegian University of Science and Technology, Trondheim, Norway; ^2^Center for Child and Adolescent Mental Health, Eastern and Southern Norway, Oslo, Norway; ^3^Regional Centre for Child and Youth Mental Health and Child Welfare North, Faculty of Health Sciences, UiT The Arctic University of Norway, Tromsø, Norway

**Keywords:** school functioning, school attendance problems, primary school, student assessment, Teacher’s Report Form

## Abstract

**Introduction:**

The present paper explores how Norwegian primary school teachers’ reason and reflect when assessing students’ general school functioning in Teacher’s Report Form (TRF), where teachers are asked to compare students of concern with what is perceived as a typical student at the same age, regarding work effort, behavior, learning capacity and mood.

**Methods:**

The teachers in the sample (*n* = 7) had recently filled out the TRF as part of their participation in The Echo Study, utilizing the cognitive behavior therapy program Emotion for sad and anxious children.

**Results:**

The results show that teachers base their assessments much on person-relative, rather than group- or age relative comparisons, e.g., what is normal for the individual student. It is also identified that a safe psychosocial environment where students feel confident speaking up and exploring new ideas, is recognized as essential or optimal school functioning and attendance. This is especially important for children experiencing sadness or anxiety.

**Discussion:**

By understanding more of how teachers reason when assessing students’ general school functioning, we can aid their efforts to identify students of concern. Poor school functioning is related to school absenteeism, and teachers are front-line professionals to observe early warning signs based on their understanding of whether a student’s behavior lies within or outside the range of typical functioning.

## Introduction

Teachers work closely with their students daily and are thus well suited to identify changes in their behavior, school performance, and attendance. However, studies have shown that teachers find it challenging to identify social and emotional difficulties in a timely and adequate manner and to know what behavior falls within the normal range or what may require follow-up and support measures (Kolko and Kazin, 1993; Marsh, 2016). Often, teachers base their judgements on common sense and experience-based knowledge when differentiating between normal, transient, and age-dependent variations in mood and behavior and more worrisome cases (Rothi et al., 2008), and they find it difficult to assess whether a student behavior lies within or outside the range of normal or typical functioning (O’Farrell et al., 2023; Van den Broek et al., 2023). This particularly applies to students with internalizing difficulties, such as anxiety and depression (Cunningham and Suldo, 2014). Because these students also have an elevated risk of developing school attendance problems (Chen et al., 2024; Finning et al., 2019a; Finning et al., 2019b) it is pivotal to identify emerging internalizing difficulties at an early stage.

School attendance problems are shaped by multiple interconnected factors, including those related to the child, parents, family, peers, school, and community (Kearney, 2008; Thambirajah et al., 2008), and refer to the challenges that arise when a child is not present at school without a valid reason (Heyne et al., 2019). As school attendance problems develop, they grow complex, the number of risk factors increase, and the student feels more and more alienated from school. It is therefore important to stop the problems before they develop to prevent an emerging downward spiral of social–emotional difficulties and school absence, where negative thoughts, feelings, and actions mutually and progressively get worse. Often, school attendance problems start with negative appraisal due to a stressor in the school environment, leading to avoidance of specific situations or classes, which in turn reinforces the negative stress and pattern of appraisal (Hancock et al., 2013; Havik and Ingul, 2021; Ingul et al., 2019).

Altogether, this demonstrates how students’ school functioning, which includes academic achievement, social and relational skills, adjustment, and behavior, all embedded within the broader context of school climate and organization (Gustafsson et al., 2010), can vary significantly across time and situations. Thus, contextual and relational factors have a major impact on the development, regulation, and maintenance of various students’ difficulties, depending on how students’ academic and social needs are met, affected, and addressed by school personnel (Bingham and Sidorkin, 2004; Hamre and Pianta, 2006; Kostøl and Mausethagen, 2011). For example, students who experience a positive psychosocial environment and good relationships with teachers and fellow students also have higher academic functioning (Haapasalo et al., 2015), and meta –analyses have identified significant correlations between the quality of student-teacher relationships and student behavior, academic performance, motivation, engagement, and well-being (Emslander et al., 2025; Quin, 2017). Students with internalizing difficulties are especially vulnerable to poor psychosocial environments and the quality of teacher-student and peer relationships are, together with academic problems, identified as key school related factors for school absenteeism (Gubbels et al., 2019). The creation of a safe environment is also identified as an important signpost for interventions to promote school attendance and prevent school refusal (Heyne and Brouwer-Borghuis, 2022), and unpredictability in learning environments and fear of negative evaluation from peers are both central school related factors for school absenteeism (Dannow et al., 2018).

In the TRF, the concept of school functioning is operationalized through the indicators of work effort, behavior, learning capacity, and mood, and overlaps much with the indicators of social and emotional competence (SEC) understood as a collective term for a range of skills related to the regulation of behavior, cognition, and emotion in interaction with others (Collie, 2020; Taylor et al., 2017) A key concept here is self-efficacy, understood as a persons’ belief in their own capacity to achieve desired outcomes (Bandura, 1978). Self-efficacy is very much formed by previous accomplishments (i.e., mastery experiences) and psychological states, but also by observing others and being encouraged by teachers or peers. Altogether, self-efficacy is vital for students’ persistence in activities, their school engagement and levels of defensive behavior. Extensive research has also documented a strong reciprocal correlation between students’ school engagement and school functioning, particularly in the sense that improving social skills has a positive effect on school performance and participation in learning activities for students with internalizing difficulties (Cordier et al., 2021; Sancassiani et al., 2015).

This paper investigates how Norwegian primary school teachers reason when assessing students’ general school functioning in Teacher’s Report Form (TRF) (Achenbach and Rescorla, 2001). TRF is a standardized, validated scoring system, commonly used to assess adaptive and maladaptive functioning in school-aged children and adolescents, and the tool is applied and tested across a wide range of national contexts (Ivanova et al., 2007). However, few researchers have considered how teachers’ reason when they assess students’ general school functioning. The primary aim of this study was therefore, based on the use of TRF as a tool, to gain greater insight into the process behind teachers’ reasoning about school functioning, compared to a typical student at the same age. Related to this, we wanted to explore how these assessments can assist teachers in identifying students with elevated risk for poor school functioning due to emerging internalizing difficulties.

We base our results on individual qualitative interviews with teachers (*n* = 7) who had filled out the TRF related to their students participation in a group-based indicative preventive intervention named ‘Emotion’. This intervention targets students in grades 4–6 who exhibit symptoms of depression or anxiety. Emotion was evaluated in a factorial, randomized trial called The Echo Study (Neumer et al., 2021). Teachers used the TRF to assess students’ general school functioning compared to typical students of the same age, through the questions: (1) how hard is he/she working, (2) how appropriately is he/she behaving, (3) how much is he/she learning (4) how happy is he/she? The variable values are as follows: (1) much less, (2) somewhat less, (3) slightly less (4) about average, (5) slightly more, (6) somewhat more, and (7) much more (Achenbach and Rescorla, 2001). This assumes that teachers have a clear idea of what constitutes normal functioning or a ‘typical student’ at the actual grade.

Numerous studies have addressed and discussed the psychometric properties of the TRF applied in different contexts internationally (Ivanova et al., 2007; Seleem et al., 2023), and in Norway (Kornør and Drugli, 2011; Stensen et al., 2023). Nevertheless, very few studies have examined teachers’ reasoning behind the assessments they apply. O’Neill and Liljequist (2002) explored the strategies teachers used to complete the questionnaire and identified eight main strategies, often used in combination. Observing students in different situations was by far the most frequently used strategy, whereas only 1.8% of the teachers in the sample used comparisons with students of the same age. This is interesting because the TRF explicitly asks teachers to use such comparisons as a basis for assessing general school functioning.

On this background, the following research questions were formulated:

*RQ1: How do Norwegian primary school teachers reason when scoring students’ general school functioning compared to a ‘typical student at the same age’ in TRF?*
*RQ2: How can such assessments assist teachers in identifying students at risk for poor school functioning and attendance?*

Based on the teachers’ use of TRF in this study, we discuss how their understanding of whether a student behavior lies within or outside the range of typical functioning is helpful to identify students at risk for school attendance problems. Although the study concerns a given Norwegian context, it contributes to new knowledge about the topic, and the results may be transferrable to other contexts or settings regarding teachers’ reasoning about characteristics of a typical primary level student and potential risk factors for poor school functioning and attendance.

## Materials and methods

### Study design

The current study utilizes a qualitative research design where data is collected through semi-structured individual interviews with teachers whose students had participated in the Echo study. During the Echo Study, teachers scored their students’ academic and general school functioning in TRF at three different measurement times: at baseline, post-intervention, and after 12 months (Neumer et al., 2021). However, based on the overall aim and research questions of the present study, we were not interested in the actual TRF scores, but in the teachers’ reasoning about what constituted a ‘typical’ or ‘average’ student in this age group, and what they regarded as warning signs for poor school functioning.

### Recruitment and sample

When recruiting the sample, we started with a list of teachers from 22 different schools in different parts of Norway, who had completed the baseline questionnaire (*n* = 58) in the Echo study. Of these teachers, 20 were randomly selected and contacted personally via an email that provided information about the study and a request to participate in an individual qualitative interview. Time frame was set to approximately 40 min. Four declined, one accepted, and the rest did not reply. Thus, we moved on to a more targeted purposive sampling (Robinson, 2014), whereby the researchers contacted (by telephone or email) the principals of schools that were nearby or whom they knew well. The principals then forwarded the information to the relevant teachers, also assuring that participation in the interview was seen as appropriate time spent, as the school had consented in teachers being interviewed after the intervention was completed. The researchers scheduled interview times with the teachers at appropriate schedules. This resulted in a total of seven digital interviews. One teacher was also recruited for a pilot interview in the autumn of 2022. This interview was not audio-recorded but contributed to quality assuring of the interview guide. An overview of the sample is presented in Table 1.

**Table 1:** Overview of the sample.

Informant	Gender	Years of experience	Teaching subjects
1	Female	5	English, Norwegian, and CRPE*
2	Male	14	Mathematics, natural sciences, and physical education
3	Male	18	Norwegian and social studies
4	Male	6	CREE*, Norwegian, and physical education
5	Female	14	Norwegian, English, and social studies
6	Female	25	Norwegian, mathematics, social studies, arts and crafts, CREE*, food and health
7	Male	7	Norwegian, mathematics, social studies, CREE*, natural sciences, and physical education

*CREE, Christian and other Religious and Ethical Education.

### Interviews and transcripts

Based on the research questions, we developed a semi-structured interview guide addressing the scoring of students’ general school functioning, including assessment of academic levels and what the teachers perceived as a typical student regarding work effort, behavior and mood, and the sources of information used for assessment. The interviews were conducted by two different researchers during November–December 2022. All interviews were conducted online, and audio recordings were made via *Nettskjema*. This is a platform, administered by the University of Oslo, to ensure safe collection and storage of research data in accordance with the national regulations to processing of personal data. The interviews were transcribed non-verbatim, meaning that most utterances like “uh” ah,” and “yeah” were removed. Moreover, some unfinished sentences and irrelevant details were not included in the final data extracts, and these passages were marked with […], whereas thinking pauses were marked with … Still, it was important to retain the information from the verbal account as true to its original form as possible (Braun and Clarke, 2006). Although some nuances may be lost in the translation from Norwegian to English, all authors of the present paper have checked and approved the included data extracts to ensure that the original meaning has been preserved.

### Thematic analysis: coding and analytical procedure

**Figure 1:**
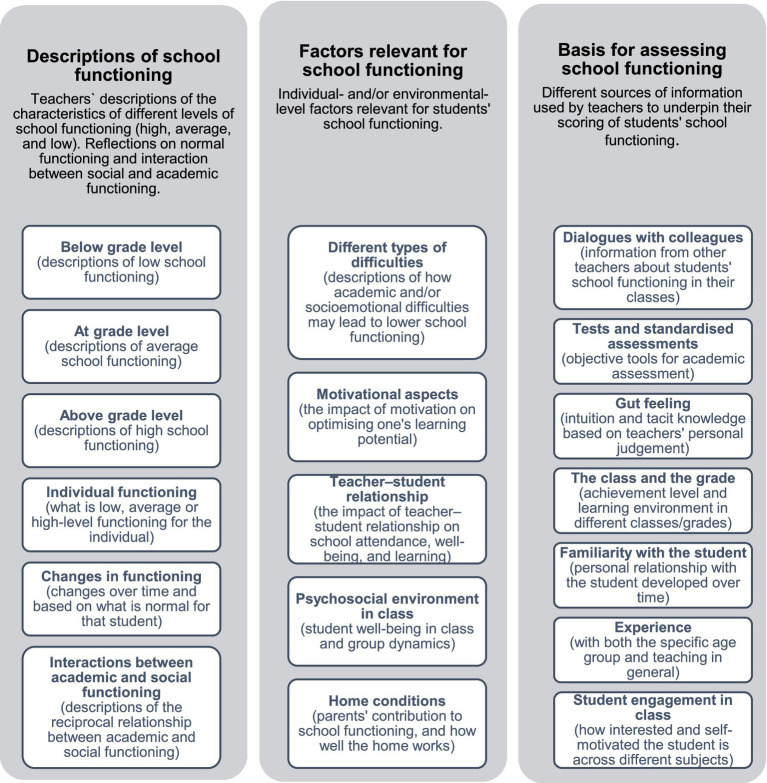
Encoding matrix with themes and codes.

As mentioned, the choice of analytical strategy affects the strength of the collected data (Malterud et al., 2015), and in our study we chose to use abductive thematic analysis combining a theory-driven, deductive approach with a more empirical, inductive approach (Thompson, 2022). This approach provides a sound structure and integrates reflexivity in the analysis of textual data derived from small samples (Mackieson et al., 2018). In the analytic process, we followed the five steps of Braun and Clarke (2006, 2022): (1) data familiarization, (2) generation of initial codes, (3) identification of themes, (4) review of themes, and (5) definition and naming of themes. Two researchers coded the material separately, and then they reviewed and revised the codes by adding, removing, and merging codes and sorting them into different themes. Regarding the question of thematic saturation, understood as the point where no new codes or themes emerge from the data, Braun and Clarke (2021) advocate the open sharing of code definitions and examples of codes and themes to improve transparency and give the reader insight into the authors’ epistemological assumptions. In the last phase, one of the researchers reviewed all codes and themes before a final matrix was prepared, which consisted of three main themes and 18 codes (Figure 1).

By developing an initial codebook including full definition of codes and thoroughly discussing and resolving any inter-coder discrepancies, we enhance the inter-coder reliability. Still, the necessity and relevance of a codebook is debated among qualitative researchers (Thompson, 2022), and instead of using absolute and written criteria for inclusion and exclusion, the researchers in our study discussed coding choices and the labelling, terminology, and definitions applied in the coding matrix throughout the coding process. It was important to avoid themes too closely resembling the topics in the interview guide while at the same time allow the codes to be guided by, but not determined by underlying theories (Thompson, 2022). Theory gives the analysis its foundation and facilitates analytic power (Braun and Clarke, 2022). Since the TRF defines general school functioning based on the four indicators of mood, work effort, behavior, and learning capacity, relative to average or typical functioning, it was appropriate for us to code the material based partly on these dimensions. However, we also introduced new data-driven codes, such as the classroom psychosocial environment, the importance of home conditions, and the relationship with the teacher.

### Validity and reliability

Validity concerns the quality of the conclusions drawn from the collected data rather than the methodological design, and validity can be threatened by, for example, overreporting how often something was said or failing to communicate important nuances in the material (Shadish et al., 2002). Construct validity is also particularly challenging because it relates to how questions are operationalized in the interview guide and the risk of construct underrepresentation or irrelevance (Kleven, 2008). The former meant that we fail to ask relevant questions about the construct of interest or ask questions that elicit much irrelevant information. Regarding reliability, the researcher effect (Cohen et al., 2017) could have been influential because the interviews were conducted by two different researchers. However, a pilot interview conducted by both researchers together, allowed us to identify potential pitfalls, and identified the room for individual adaptation in the semi-structured interview guide (Qu and Dumay, 2011). The themes included in the final guide were: (1) professional experience, background, and current work situation, including experience with students of different ages and subject backgrounds; (2) scoring of students in different subjects, including the sources of information used, why students were considered far below or above average, and what individual or environmental factors that may contribute to poor school functioning; and (3) scoring of students’ general school functioning, including what the teachers perceived as a typical student regarding work effort, behavior and mood, and the sources of information used for assessment. We also asked the teachers about what they regarded as warning signs for poor school functioning.

### Compliance with ethical standards

One of the authors of this paper is the national project leader of the Echo study, and several other authors have been involved in the design and implementation of the study. Still, none of the authors have economic or commercial interests in the intervention applied in the Echo study or in the TRF. The Regional Ethic Committee (REC) approved the Echo study (ref. no. 28761), and this additional qualitative study was also approved by the Norwegian Agency for Shared Services in Education and Research (SIKT). Informed consent from the teachers was obtained in accordance with the Declaration of Helsinki and the Guidelines for Research Ethics in the Social Sciences and the Humanities. Since the teachers did not refer directly to individual students or third parties but only gave information about their general reasoning when filling out the TRF, no sensitive data were gathered, and little ethical risk was associated with the material.

## Results

The results are presented across the three main themes identified in the coding matrix (Figure 1), to answer the two main research questions of the study. The informants are referred to as i1, i2, etc. based on the sample description in Table 1.

### Assessments of typical school functioning

The informants broadly agreed that all students in some ways were ‘typical’. One teacher explained, ‘In my eyes, they are all typical, even though they are different in their own ways’ (i6). Related to this, the informants expressed that what is regarded as typical or normal for the individual is not so easy to generalize to other students as ‘every child has his or her normal’ (i1), and that even if some students struggle more than others, ‘one must look at what is good effort for *them*’ (i1).

For academic assessment, the teachers often found it hard to identify poor academic functioning in less theoretical subjects like social studies and physical education. In the absence of standardized tests, it is much up to the teachers’ personal judgement or what some of them called ‘gut feeling’. Here, knowledge of the individual student was regarded as an important basis for assessment: ‘I feel like you could base it on. almost nothing, because you know them after a couple of—or 3 years’ (i2), and ‘it is really not that difficult to make assessments when you know the students so well’ (i6). This personal knowledge also weighed heavily if different teachers had different opinions of the students: ‘I think that most teachers know their students well enough to ultimately trust their own gut feelings and opinions’ (i1). Other teachers said much of the same and added pervious experiences with the age group as important bases for assessment: ‘It is an overall assessment based on experiences I have had with the student group and previous experience with groups of students of the same age’ (i7) and ‘it becomes an average standard that I kind of make in my own head’ (i3). When the teachers described what they regarded as a typical student’s learning capacity and work effort, based on age, it largely concerned the student’s independence, self-motivation, and academic comprehension in working on assignments in class:

It’s kind of hard to say … what a typical student is. It’s tempting to say that’s the ‘dream student’, right? But that’s not it. I think that a typical student […] you expect a sixth grader to be able to take a message and sort of do what is expected of them without too much guidance. (i1).

Regarding mood, the teacher informants described typicality here as the mood being generally steady, with certain fluctuations due to the student being tired or unfocused. However, like informant 2 said, it is not normal to ‘be unfocused all the time’. They also found it completely normal that students in grades 4–6 increasingly show that they find schoolwork boring.

### School-related influences on school functioning and attendance

The teachers commonly agreed that the class environment is highly important for academic performance. One teacher says: ‘It reflects everything. The class that I have now has a much better class environment, better behavior, and they perform much better as well’ (i2). Another teacher said:

Class environment is really important. And at this school, the current sixth grade we have is divided into A and B, and in there we have certain groups that are a bit stronger, in a negative sense. We’ve rotated the classes a bit, because those who are very good… they sort of end up in the background. They do not really dare to show themselves that much. Because there are others who take up more space. If a student comes to school and is anxious about speaking in class or anything like that, because of their peers, I cannot see it as anything other than negative. (i3).

A similar reflection was shared by a third informant:

The class environment, I think, is of great importance. A safe environment where it’s okay to answer incorrectly, or okay to jump into something you are a little unsure about and give it a try. I think this has a big impact on whether someone scores below average’. (i7).

Furthermore, the teachers discussed the problem of narrow range of activities, limiting the students’ opportunities to show their competence. Moreover, since active engagement as an expression of effort is often used as a basis for assessing academic performance, the teachers also mentioned that it could be both inhibited or facilitated by the class environment in terms of how safe it is to speak up and dare to try new things.

The teachers also reflected upon how difficulties related to motivation and concentration can prevent students from realizing their potential and functioning optimally:

A lot of other things happen during breaks and things like that, and they cannot keep up when mathematics starts in class. They sit afterwards and say they do not understand anything. Then, their attitudes become very like: ‘But I cannot do this.’ It’s because they do not put effort into it. […] They do absolutely nothing. They opt out instead of trying to do it right. (i5).

Related to this, the informant expressed a fear that such behavior may create a vicious cycle in which the students stop believing in their abilities, make less effort, fall behind, and eventually start skipping classes. She believes that this problem is more common among girls because they more often set a personal standard for themselves to “understand everything and know it all” (i5). Another teacher also talked about this and reflected upon why some students develop what she calls “lesson specific refusal,” representing systematic non- attendance at lessons with subjects they find difficult, for example math (i1).

### Reasoning about causes of concern

The teachers recognized that there may be many reasons for why students perform below their potential. One teacher said:

We do have some students who, perhaps initially, are placed at or above average, but for various reasons choose not to work, and as a result, in a way, score lower or start to fall behind because they either do not want to or cannot work for other reasons. We do have cases of this (…) A bit of underperformance. For different reasons. Some do it because they have difficulties at home. Some do it because they have difficulties with themselves. Some just have not cracked the code, so to speak (…). (i1).

Examples of such “various reasons” were given also by other informants, where especially home conditions were referred to as the ‘cornerstone’ (i2) and the ‘pillars’ or ‘scaffolding support’ (i7) for students to function well at school. One teacher, for example, said that students may have problems absorbing knowledge because ‘the focus is elsewhere. Maybe they bring problems from home that we know nothing about’ (i6). Another teacher also reflected upon the importance of taking home conditions into consideration when understanding students who ‘might be a bit outside of the norm socially and in terms of behavior and such’ (i2).

Finally, the teachers made the point that general school functioning needed to be assessed and observed over time, to separate between random events and more enduring patterns of changed behavior:

But of course, when you see that a student suddenly has fewer friends or is always alone, or when no one interacts with them during class—they just sit there. Then you start to think. And this can happen over time. If there’s an argument and someone is upset, it might last for a day, and then things go back to normal. But when you see it happening for weeks, then. (i2).

Sudden changes in behavior were also identified as a major cause of concern:

But it is also when we see increasing restlessness among students. I mean, if we notice that they start deviating from their usual behavior, the behavior that is normal for them I. […] For example if a typically calm student suddenly begins to respond rudely and arrive late to class. Yes, you notice a bit of school avoidance or more of a class avoidance, right (.) if you have students who previously managed to stay focused, but they start becoming distracted and restless. And they show a negative change in behavior. (i1).

### Summary of findings

To sum up, most teachers were keen to convey that they generally regarded all students as ‘typical’ or ‘normal’, and their reasoning centered much around person-relative comparisons (i.e., what was characteristic of the individual). These assessments required good personal knowledge of the student and the ability to observe changes in behavior and academic performance over time. However, the teachers also relied much on previous experience with students of the same age, and made some average standards based on this. Finally, they considered environmental factors (e.g., psychosocial class environments and home conditions) as highly important for students’ school functioning and learning capacity.

## Discussion

A prominent finding in our study was that most of the teachers highlighted the importance of assessing students’ general functioning based on the students´ own ‘normal’ and what is typical for the individual. This aligns with O’Neill and Liljequist’s (2002) findings showing that only 1.8% of the teachers in their sample used comparison with students of the same age as the main strategy for scoring general school functioning in the TRF. Nevertheless, the teachers shared some common beliefs about what behavioral shifts are normal for students of that age, including concentration, mood, and work effort. They also corrected themselves, stating that a ‘typical student’ is not the same as a ‘dream student’. For learning capacity and work effort, the teachers placed great emphasis on students’ ability to work independently, and they assessed typical functioning based on the students’ self-motivated problem-solving. This is related to the students’ self-efficacy and the levels of work effort and persistence in the face of obstacles (Bandura, 1978). According to Gustafsson et al. (2010), internalizing problems such as depression and anxiety may cause students to view their own capabilities in a more negative manner and thus may also affect their self-efficacy and academic confidence. Low self-efficacy for academic coping is also characteristic for students with school attendance problems (Ingul et al., 2019). Addressing the potential underlying causes of poor effort is therefore important to facilitate school functioning.

The teachers seemed to recognize that students in grades 4–6 gradually become less motivated for and engaged in schoolwork, and more explicitly express their boredom. However, the *frequency* of behaviors like being unfocused, angry or sad, seems to be perceived as a key characteristic for identifying less typical school functioning and help teachers identify students who might benefit from additional support regarding their SEC (i.e., their ability to regulate their behavior and emotions in social interaction) (Collie, 2020; Taylor et al., 2017). Improving social skills has also been identified as having a positive effect on school performance and engagement in learning activities for students with internalizing difficulties (Cordier et al., 2021; Sancassiani et al., 2015). Internalizing difficulties are found to be a true risk domain for school attendance problems and should be considered when assessing school functioning (Finning et al., 2019a; Finning et al., 2019b; Gubbels et al., 2019; Ingul et al., 2019). Thus, adequate identification of students’ psychosocial problems is required to provide sufficient training of social and emotional skills, which in turn may contribute to better school functioning, belonging and school attendance.

Moreover, academic problems and poor psychosocial environments with low quality of teacher-student- and peer relations, may also be a risk factor for school attendance problems (Gubbels et al., 2019). A positive psychosocial environment is therefore very important, and our study revealed that the teachers were highly aware of how the classroom environment could inhibit or promote students’ participation in learning activities. The teachers were also aware of their own role, recognizing that insufficient support in the classroom could hinder students from reaching their full potential. This finding is supported by previous research indicating that contextual and relational factors have a major impact on the individual student’s school functioning (Gustafsson et al., 2010; Hamre and Pianta, 2006; Kostøl and Mausethagen, 2011). An unsafe environment in which students are afraid of speaking up or making mistakes reinforces students’ existing internalizing difficulties and withdrawals. For some students, this can also hinder help-seeking behavior, such as asking questions when they do not understand, which in turn contributes to reduced learning outcomes and lower academic functioning and attendance.

Moreover, our results indicate that a narrow range of activities in the different subjects may limit the students’ opportunities to show their competence. To broaden educational options and adjust educational tasks is identified as a core factor to promote school attendance (Dannow et al., 2018; Heyne and Brouwer-Borghuis, 2022). Maladjusted academic levels may also reinforce a tendency towards underperformance such as lack of effort or actively avoiding lessons in certain subjects. Several teachers in the study identified this as both a potential cause *and* result of low academic functioning and talked about a vicious circle of skipping lessons the students find difficult, which in turn makes it even more difficult for the students to keep up with the subject. This negative pattern is highly important to address at an early stage, to prevent school attendance problems from escalating (Hancock et al., 2013) and shows how assessment of school functioning is relevant for identifying students at risk for school attendance problems. Children struggling with school refusal or subject specific refusal are often not properly identified with their emotional difficulties and can be misinterpreted as lacking motivation (Thambirajah et al., 2008). Therefore, it is important that the teachers have sufficient time and opportunity to get to know their students, academically, emotionally and socially, enabling them to better understand the underlying reasons for non-attendance.

Our findings indicated that experience with the age group, overall teaching experience, and knowledge of individual students were all factors that the teachers in our study highlighted as decisive elements to assess school functioning. However, sustained misbehavior, and sudden changes in behavior and mood were both evoking concern among the teachers, and to identify this, they needed to know their students and observe them over time. This resonates with the findings reported by O’Neill and Liljequist (2002), where teachers very much rely on their experience with and observation of students in different settings and interactions, when assessing their school functioning. Hence, these factors may also be relevant when it comes to the validity of their TRF scores, since the TRF also asks teachers to rate how well they know the student (not well—moderately well—very well). Thus, the relevance of TRF as a tool for identifying students at risk for poor school functioning and attendance, depends much on the teachers’ familiarity with the student.

The teachers also recognize the importance of family support and talk about the home as the ‘pillar’ or ‘cornerstone’ for students’ school functioning. They recognize that problems at home can make it difficult for students to focus on class, and in turn contribute to subject-specific non-attendance or underperformance. Altogether these findings illustrate the complex interrelations between individual, school and home factors in school functioning and attendance as discussed by Kearney (2008), and how systemic thinking is useful to address and understand these relationships. A key implication for practice is that raised awareness on how teachers reason about typical school functioning, is vital to identify students’ needs for additional support and thereby also reduce the risk for later negative trajectories and school attendance problems.

### Limitations

The present study has some limitations. First, seven informants are modest, and we may have achieved richer data with a larger sample. Whether a sample of seven participants can provide sufficiently strong data depends on the purpose of the study, the quality of the interview dialogues, and the analytical strategy chosen (Malterud et al., 2015). In our case, the quality of the interview dialogues varied somewhat depending on the comprehensiveness of the answers the informants gave, but we achieved suitable breadth of information regarding the informants’ teaching subjects and professional backgrounds. Also, they had all filled out the TRF recently, which made it easier for them to recall their reasoning. Thus, we argue that satisfying information power was gained, and the collected data enabled us to address the research questions of the study. Nonetheless, we are aware that the shift of recruitment strategy from random to purposive sampling may represent a potential selection bias, and reflexivity in the analytical process is therefore highly important to strengthen the rigor and mitigate the bias. Thematic analysis is regarded as a highly suitable approach in this regard (Mackieson et al., 2018). Second, the small sample size may limit the generalizability or transferability of the research. However, since the informants represent different teaching subjects and school settings (i.e., urban and rural areas), their perspectives and experiences are likely to be relevant to the broader school community. Still, this transferability may be both an inductive and deductive process, where the first represents an abstraction of data obtained from a specific sample, setting and time to more general insights, and where the latter represents application of these insights to new situations and people (Drisko, 2024). Third, web-based interviewing represents a risk of some aspects of communication getting lost. Still, online methods are more flexible, convenient, and cost-effective, and may also be preferred over in-person interviews by the participants (Archibald et al., 2019). Fourth, there is a risk of interviewer-bias, where the wording of the questions or the interviewer’s behavior can lead to an interviewee’s response (Cairns-Lee et al., 2022). By using two interviewers and reflecting on our potential different impact on the interviewees, we mitigate this potential bias. One of the interviewers was directly involved in the development and implementation of the Echo study, whereas the other was more peripheral. This facilitated researcher reflexivity and provided a good basis for discussing inter-coder discrepancies in the analytical phase. Finally, overreporting and lack of nuance in reporting the findings are common threats to validity (Shadish et al., 2002). Therefore, we paid careful attention to include quotes from a good breadth of informants, and to highlight both similarities and differences in the informants’ views and experiences.

## Conclusion

Poor school functioning is a risk factor for school attendance problems, and we know that both academic and emotional difficulties play a role in this. How teachers reason about the causes of poor school functioning, and what is perceived as a deviation from typical or normal functioning, is therefore important in order to help students function better in their everyday school life and to prevent unwanted absence. Since the TRF is a widely used tool for identifying pupils who show signs of emerging or established difficulties related to school functioning and behavior, it is important to understand the reasoning teachers apply when assessing pupils in these areas. This, in turn, can form the basis for better prevention of school absenteeism, by gaining a better understanding of the teacher’s perspective on what characterizes and contributes to good school functioning.

The main takeaway message from the current study is that teachers´ understanding of age-based typicality is quite variable. First, teachers tend to base their assessments of general school functioning much on person-relative, rather than group-relative comparisons, e.g., what is normal for the individual student regarding work effort, behavior, learning capacity, and mood. Based on this actual use of the TRF, teachers’ ability to identify students at risk seem to rely more on how well they know the student and what is normal for the individual, rather than on what they know about normal functioning for the age group. Thus, the teachers’ familiarity with the student, and their ability to observe the student over some time and in different contexts, may strengthen the validity of the assessments and enhance TRF’s role as a potential aid in identifying students at risk for poor school functioning and attendance. Second, teachers paid strong attention to the psychosocial environment in class, and how this may facilitate or inhibit anxious and/or sad students’ school functioning through the provision of opportunities to show active engagement and dare to fail. This insight is important to aid teachers in their efforts to assess and understand students with school attendance problems, and to put focus on the role and mutual relationship between individual, school and home factors in school functioning and school attendance.

## Data Availability

The datasets presented in this article are not readily available because the dataset is only available in original Norwegian language. Requests to access the datasets should be directed to stine.m.ekornes@ntnu.no.
